# Groundhog Day in the emergency department: A systematic review of 20 years of news coverage in Australia

**DOI:** 10.1371/journal.pone.0285207

**Published:** 2023-05-02

**Authors:** Elizabeth E. Austin, Nadia Lanos, Karen Hutchinson, Susan Barnes, Diana Fajardo Pulido, Colum Ruane, Robyn Clay-Williams

**Affiliations:** 1 Australian Institute of Health Innovation, Macquarie University, Sydney, New South Wales, Australia; 2 School of Social Sciences, Macquarie University, Sydney, New South Wales, Australia; 3 General Education Department, City University of Macau, Macau, China; Western Sydney University / University of Melbourne, AUSTRALIA

## Abstract

This study examined how the Australian news media have portrayed public hospital Emergency Departments (EDs) over the last two decades. A systematic review and media frame analysis, searching Factiva and Australia and New Zealand News Stream for digital and print news articles published between January 2000 and January 2020. Eligibility criteria were (1) discussed EDs in public hospitals; (2) the primary focus of the article was the ED; (3) focused on the Australian context; (4) were published by one of the Australian state-based news outlets (e.g., The Sydney Morning Herald, Herald Sun). A pair of reviewers independently screened 242 articles for inclusion according to the pre-established criteria. Discrepancies were resolved via discussion. 126 articles met the inclusion criteria. Pairs of independent reviewers identified frames in 20% of the articles using an inductive approach to develop a framework for coding the remaining articles. News media rely heavily on reporting problems within and with the ED, while also proposing a cause. Praise for EDs was minimal. Opinions were primarily from government spokespeople, professional associations, and doctors. ED performance was often reported as fact, with no reference to the source of the information. Rhetorical framing devices, such as hyperbole and imagery, were used to emphasise dominant themes. The negative bias inherent in news media reporting of EDs could potentially damage public awareness of ED functioning, with implications for the likelihood of the public’s accessing ED services. Like in the film Groundhog Day, news media reporting is stuck in a loop reporting the same narrative over and over again.

## Introduction

The way news is consumed in Australia has changed over the last two decades. Between 1996 and 2000, news services were moving online, led by the Australian Broadcasting Company (ABC), which was the first Australian site to combine international, national, state, and regional news in text, audio, and video formats. In 2008, the first iPhone was released in Australia, making the news even more accessible [1]. News is an important source of information for communities, including information about public health and health care services such as Emergency Departments (EDs) [2]. EDs across Australia are increasingly crowded because of rising demand, longer wait times and overall lengths of stay leading to poor patient outcomes [3]. Given the essential role EDs play in providing access to urgent health care and the power of news in shaping consumer perceptions and behaviour, it is important to examine how EDs are portrayed in digital and print news media. Understanding the intricacies of how the media portray EDs in news articles can provide critical insight into how public opinion may be constructed through frames and discursive strategies (i.e., journalistic techniques). This task is particularly important in today’s media environment of misinformation and the reporting of increasingly polarised content.

News media are an important vector of information for communities about public health, exemplified by the current reliance on daily press conferences and live blogs for information about the COVID-19 pandemic. While news media are often portrayed as objective reporting of the facts, the media actually limit or define meaning and shapes perceptions by selecting what to include and exclude from a story [4, 5]. Existing research has examined how a broad range of public health issues have been framed by the digital and print news media–e.g., illicit drug use [6, 7], alcohol consumption [8], complementary and alternative cancer treatments [9], genetic medicine [10], mental health and suicide [11–19], swine flu [20, 21] and EDs [22, 23]. These studies found that foregrounding information creates or frames a unique narrative for the reader. These frames then become a powerful mechanism that shapes public opinion and behaviour. With 291 public hospital EDs and 41 private hospital EDs across Australia, news frames have the potential to influence when people choose to present to an ED, including decisions to delay accessing care, as well as which ED they will attend or bypass [24, 25]. Therefore, examining how digital and print news media frame healthcare services like EDs is important for understanding communities’ perceptions and use of the service.

### News frames

Journalists frame information by selecting an aspect of a perceived reality and communicating it in a way that makes it more salient, ultimately, promoting it [26]. News journalists predominantly shape and direct the narrative by using the discursive strategies of language, rhetoric, and journalistic practice. The intentional use of framing devices such as metaphor, exemplars, catchphrases, depictions, and visual images are used to assemble a narrative to promote a particular interpretation of an event, topic, or person [5, 21]. For example, Blood et al. [12] explored framing strategies of news reports on mental illness that revealed numerous instances in which journalistic choices of sources, metaphor, and language emphasised narratives that connected mental health issues with violence. Special attention was placed upon the headline and leading paragraphs as these foregrounded the position the author chose to take. Similarly, Petersen [10] conducted a discourse analysis of how “news media ‘frames’ stories on genetics and medicine” (pp.1258). The study showed how catchy headlines, expert sources, and metaphors were used to prime readers, and foreground dominant narratives (i.e., frames) of hope and discovery surrounding genetic research and medical developments. Through discursive strategies (e.g., catchy headlines, expert sources, and metaphors) journalists packaged information into frames to represent and/ or reflect the aspect of reality they sought to portray [10]. Consequently, the discursive strategies contemporary news media use to frame reality or how a news story is presented to the public may play an important role in shaping social biases and assumptions.

### Sources of information

News media is one source for consumers to access information about the health system. However, the Australian public also has access to ED performance data published by state and federal government health organisations. For example, the MyHospitals website was developed by the Australian Federal Government and launched in December 2010 to provide access to public hospital performance information [27]. In 2011, public reporting of government-funded (public) hospital performance data was mandated. The National Emergency Access Target (NEAT) was piloted in Western Australia in 2009 and progressively implemented across Australia, in response to evidence that ED crowding and increasing lengths of stay were resulting in adverse events [28]. NEAT defined hospital targets for the proportion of patients admitted or discharged from ED within 4 hours of presentation. While NEAT ceased being a Commonwealth target during 2014–2015, states continue to collect and report information about ED performance, including access targets and time to treat. In 2017, the Australian Government Department of Health started publishing media and ministerial releases, primarily on flu vaccines, childhood immunisation, and medical exemptions. While the Australian public has access to government data websites, these information sources require the ability to search and synthesise data (often critically sifting through a lot of information), whereas news is already pre-prepared into small digestible packages (the ‘fast food’ of information) which is easier and faster to access.

While digital and print news media are not the only sources of information about EDs, digital and print news media is typically more widely and easily accessed by the general public, and therefore more influential in shaping public perceptions and health-seeking behaviours than government reports [2, 29, 30]. Critically, there has been limited examination of the coverage of EDs in Australia. Kennedy et al. [22] reported the newspaper articles published between 2003–2004 negatively portrayed EDs through their focus on problems and errors combined with political and bureaucratic commentary. Instead, research has examined media representation of other public health issues such as alcohol consumption [31], processed food intake [32], and vaccination [33]. While research shows that readers perceptions of responsibility of health problems such as obesity and diabetes are not influenced by sources of information [34], information framed to emphasise benefits tend to be more persuasive for low-risk actions and information framed to emphasis cost are more effective for high-risk actions [35]. For community members, a delay in accessing ED care may be a high-risk action. A comprehensive examination of how the Australian digital and print news media portray EDs is particularly pertinent considering that EDs are an important point of entry to the hospital system for the public.

The way news is accessed and consumed has changed over the last two decades. News is now easier and cheaper (often free) to access. The shift from paper-based access to online, and adoption of the 24-hour news cycle, coupled with increased volume and automation of reporting, and less monitoring and regulation, means news stories are more compact, more frequent, and more easily accessible and shareable via social media such as Facebook [36]. As such, information, including news, shared on Facebook has the potential to shape health behaviours [36]. Critically, news on social media is not effectively filtered and can result in the sharing of misinformation and fake news (i.e., fabricated information that mimics news content in form) [36]. These changes have elevated the potential for misinformation to slip through. The spread of misinformation is promoting erroneous practices that result in poor health outcomes [37]. While objective sources of online healthcare reporting, such as Croakey Media and The Conversation are emerging in response to reported misinformation, these would not yet be considered mainstream. It is important, therefore, to examine how journalists frame health services like EDs in newsprint. To do so, we examined how the Australian digital and print news media portray public hospital EDs by studying news stories about public hospital based EDs in Australia. For this study, the term ‘print media’ has been used to refer to the traditional and online news media–newspapers and newspaper websites–which publish news articles. Thus, we pose two research questions:

RQ1: How has Australian news media presented information about public hospital EDs prior to the COVID19 pandemic?

RQ2: How has the Australian news media reporting changed over the two decades prior to the COVID19 pandemic?

## Methods

We conducted a qualitative study using existing and publicly available data sources. In Australia, news stories are reported across multiple platforms including print, radio, and television. Given that stories are often repeated across the different mediums, stories published in print was used to define boundaries around the data under analysis. Given that the shift from primarily print to online platforms began in 2000, we conducted a review of published digital and print news media over the last 20 years (from January 2000 to January 2020) related to EDs. To capture articles available to the wider Australian audience, our review included articles from national and state-based newspapers.

While best practice states the data should be updated every 6 months [38], articles and information post-January 2020 were deliberately excluded to avoid introducing bias through coverage of the COVID-19 pandemic.

### Inclusion criteria

Digital and print news media articles published in the English language were included that met the following additional criteria: (1) discussed EDs in public hospitals; (2) the primary focus of the article was the ED; (3) focused on the Australian context; (4) were published by one of the following state-based news outlets: The Australian, The Guardian, The Sydney Morning Herald, The Daily Telegraph, Herald Sun, The West Australian, The Courier-Mail, The Advertiser (formerly The Adelaide Advertiser), The Age, and the ABC. These outlets were selected based on national coverage and national distribution.

Articles were excluded if: (1) they were not news articles, such as editorials, letters to the editor, media releases (i.e., official statement to provide information to the public and media published on government websites); (2) discussed EDs in private hospitals; (3) discussed EDs in other countries; (4) the primary focus of the article was not the ED; (5) the focus of the article was a specific event, case or communicable disease.

### Search strategy

To identify eligible articles, we developed a comprehensive search strategy using keywords for the general concepts of ED. Factiva and Australia and New Zealand News Stream were searched on 15 January 2020. A date limit of 1 January 2000 was set to capture news articles published in the last 20 years. The full search strategy for both databases was as follows:

“Emergency Department” OR “ED” OR “Emergency Ward” AND “Hospital”

### Study selection

The results of the searches were entered into a Microsoft Excel spreadsheet and duplicates were removed. For each article, title and full-text were independently screened by a pair of reviewers (NL, EA) for inclusion according to the pre-established criteria outlined above. Disagreements were resolved via discussion.

### Data processing and analysis

The dataset was analysed using news media frame analysis as a conceptual tool for thinking about underlying beliefs, values, and priorities at play in ED news media discourse. Frames provide structure and boundaries by defining ideas and categories, capturing meaning embedded in news [26]. Using pre-existing frames, such as those identified by Neuman, Just [39] (e.g., human impact, powerlessness, economics, moral values, and conflict), allows for the generation of specific hypotheses about the portrayal of EDs in the Australian context. While inductive approaches to identifying frames rely on a small sample of articles to construct frames from news content, inductive approaches allow for greater flexibility in frame identification, aligns more closely to the content, and supports the generation of new understandings about media narratives of EDs in the Australian context [40, 41]. Therefore, an inductive approach was adopted to identify frames.

Five ED articles were independently coded by the team of seven researchers using NVivo12 software [42] to identify initial frames and subframes. See S1 Table for the full list of analysed articles with the five articles that contributed to the initial identification of frames and subframes highlighted. Codes were synthesised and categorised through discussion, and keyframes and subframes were used to develop an agreed framework for coding the remaining ED articles. Twenty percent of ED articles were then independently coded by the seven research team members using the agreed framework. Through an iterative approach, additional frames and subframes that arose from independent coding were discussed, synthesised, and incorporated into the framework in team meetings. Frames were categorised in all ED articles by pairs of independent reviewers. When differences arose, pairs discussed their reasoning. When the pair could not resolve the difference, the difference was raised for discussion in a team meeting. Differences were resolved by returning the focus of the research questions and the shared understanding of the framework. No new themes emerged from the coding of the remaining 100 articles. See S1 Appendix for an example article with frames highlighted.

## Results

**Fig 1:**
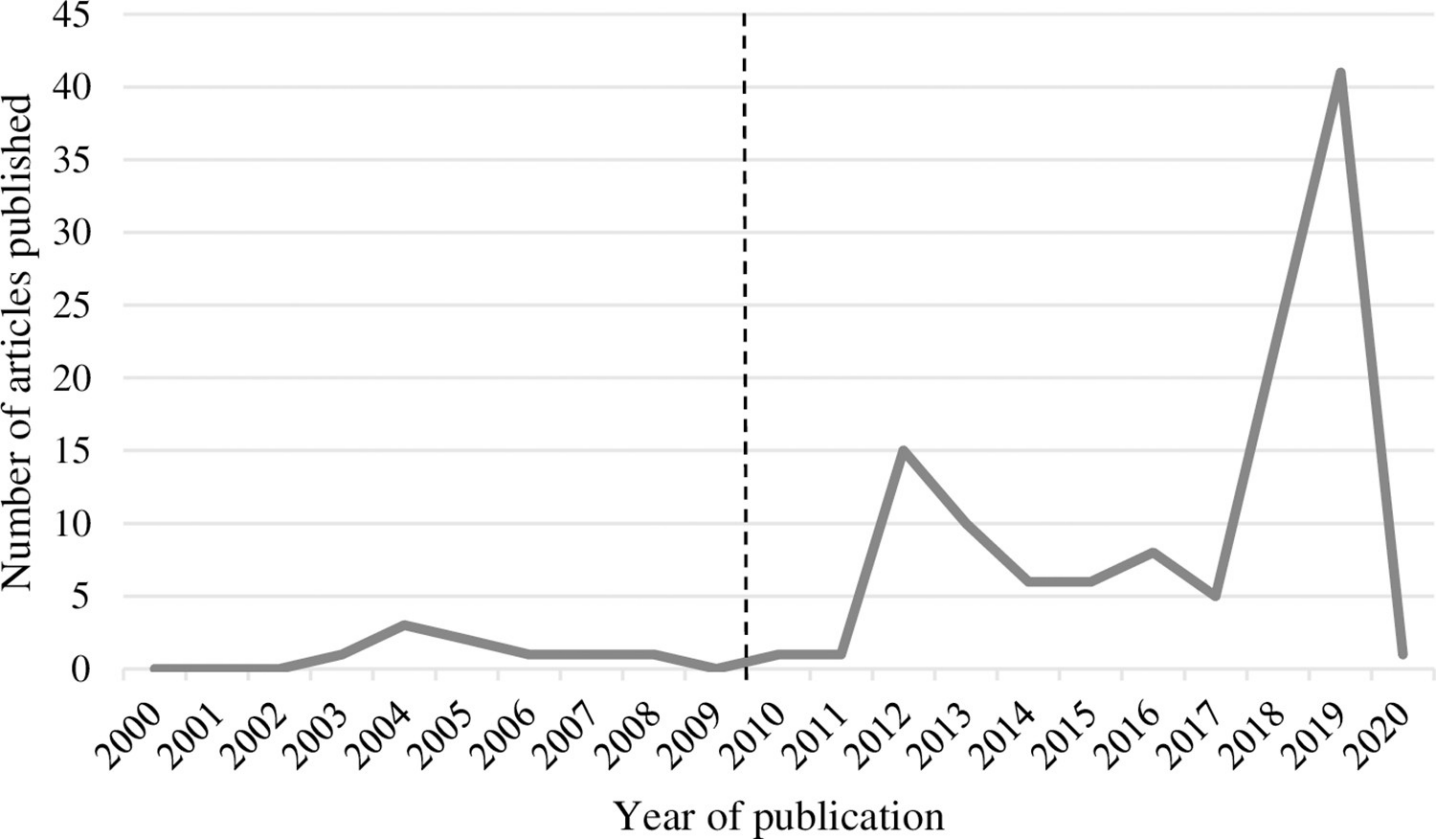
The number of ED articles published per year. The dotted line indicates when NEAT (i.e., the four-hour rule) was introduced across Australia.

**Fig 2:**
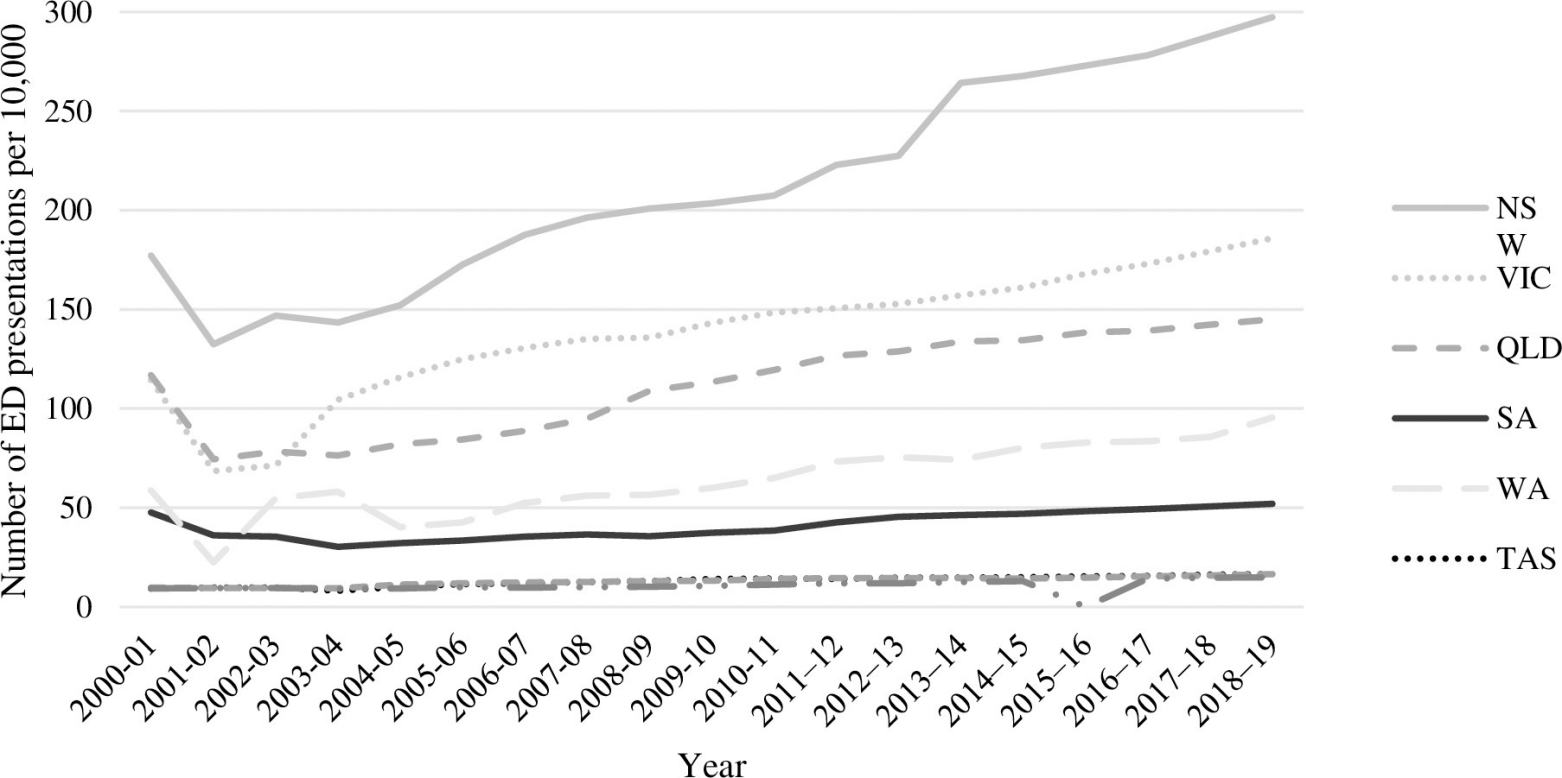
The number of ED presentations per 10,000 per year.

**Fig 3:**
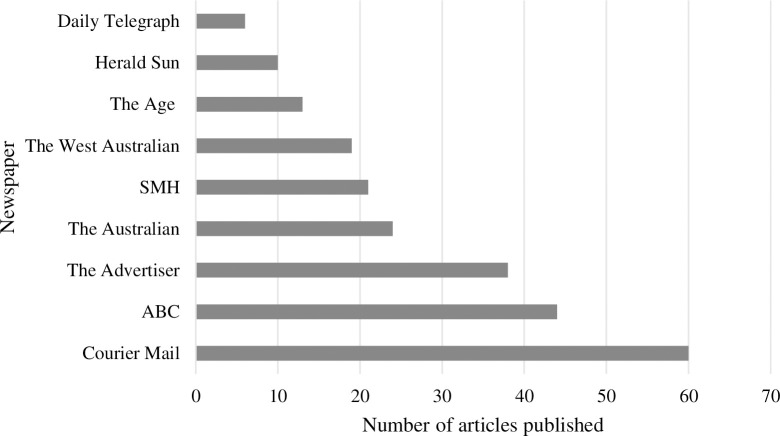
The number of ED articles published per newspaper over the study period.

The combined searches yielded 242 articles. Of these, 126 articles met the inclusion criteria. Between 2000 and 2011, coverage of ED in news media was minimal (i.e., 0–3 articles per year). Between 2012 and 2020, coverage of ED increased to 5–41 ED articles per year. The greatest number of ED articles published in any one year was in 2019, with almost 33% of included ED articles published that year. Fig 1 presents the number of ED articles published per year over the study period. Fig 2 presents the number of ED presentations for each state or territory per 10,000 population per year. From 2012 there was a gradual increase in articles, with a marked increase from 2018 onwards. Of the whole dataset, 60 ED articles (48%) were published by The Courier-Mail and 44 (35%) by the ABC (see Fig 3).

Six frames were developed from the data and incorporated into our coding framework: (1) The ED system is full of problems; (2) The causes of problems are varied and interdependent; (3) Solutions can improve ED care delivery, but few are implemented; (4) ED systems are difficult to praise as they don’t often meet efficiency performance targets; (5) The opinions and assertions of patients are undervalued compared with bureaucrats; and (6) Sources of information about the ED can increase the perception of poor ED performance. Discursive strategies were also explored from the data to answer the research questions.

### Frame 1. The ED system is full of problems

Problems within and with ED services were given high visibility, particularly between 2012 and 2020, with 85% of ED articles mentioning a problem. Problems included:

- avoidable adverse events (e.g., patient deaths),
- violence against frontline health workers,
- infrastructure not meeting service demand (e.g., insufficient beds, technology functionality),
- hiding and/or denying problems exist (e.g., creation of short-stay units to disguise wait times, data/statistics misrepresents ED functioning, falsification of data),
- the types of conditions patients present to ED with (e.g., intoxication, GP or pharmacist treatable conditions, mental health),
- workforce shortage (e.g., inadequate staffing, extended on-call hours, lack of cultural diversity), and
- the length of wait times (e.g., ambulance ramping, over 4 hours extending into days waiting in ED).

**Fig 4:**
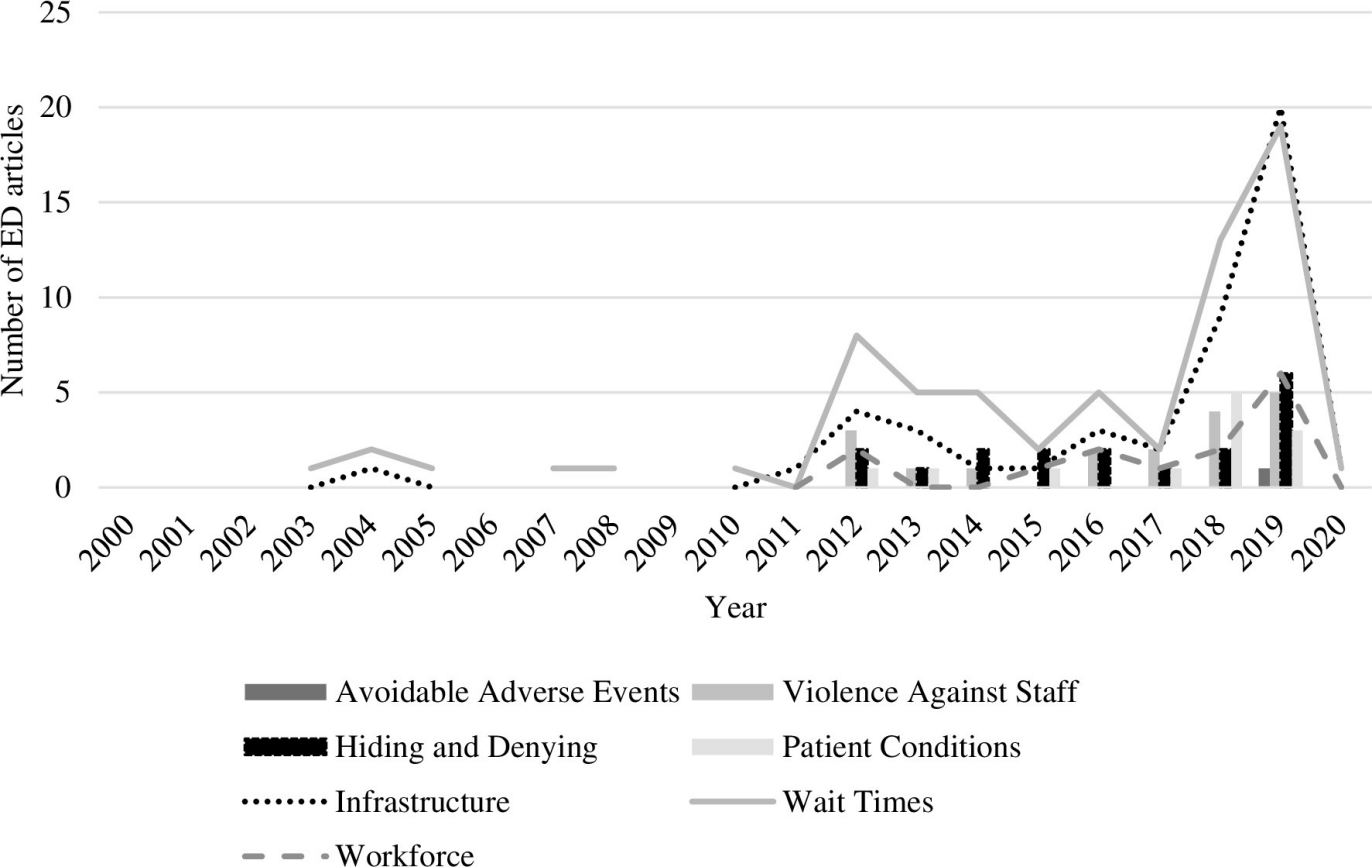
The number of ED articles mentioning problems by type from 2000–2020.

Of the seven identified problems, infrastructure and wait times were given the highest visibility, with both peaking in 2019. Wait time especially is shown to be a frequently highlighted problem as compared to the other issues, with it consistently reported more than any other problem from 2011 to 2019. Problems relating to the adequacy and appropriateness of treatments (e.g., patients shackled in corridors, delays in patients’ treatment for acute conditions) were also reported. Problems were reported in clusters as a chain of circumstances. For example, mental health patients undergoing long wait times because infrastructure not meeting service demand can be associated with increased violence against frontline workers. Fig 4 presents the publication of ED articles mentioning the different problems over the two decades.

ED problems were often described in terms of performance perceptions, e.g., “*The hospital has the second busiest emergency ward in the state and consistently records some of the worst wait times for treatment*” [43]. Furthermore, journalists tended to provide an extensive description of insufficient beds in hospital wards, technology malfunctions, and ambulance ramping associated with EDs capacity to cope with service demands: “*Critically ill patients are treated on trolleys in the hospital’s corridor or in the back of ambulances because of a lack of beds in the at-capacity hospital*” [44], “*Integrated Electronic Medical Record system crashed at every one of the 14 hospitals*” [45], and “*Ambulances were being used as overflow wards*” [46]. In contrast, the depiction of avoidable adverse events due to the “*increasingly unsafe work environment*” [47] and workforce shortage was minimally described across ED news articles by journalists: “*there has been an almost 50 percent increase in patient numbers*, *but almost no increase in doctors since 2008*” [47] and “*death linked to inadequate staffing*” [47].

### Frame 2: The causes of problems are varied and interdependent

Causes of problems within and with ED services were also given high visibility, particularly between 2017 and 2020, with 80% of ED articles proposing a cause. Causes included:

- patients’ characteristics (e.g., race, age, health condition complexity),
- hospital-related (e.g., insufficient infrastructure, design or operation, management, workforce),
- community-related (e.g., population growth, insufficient community health service availability, ambulance service functioning),
- the government (e.g., insufficient funding, political commitment), and
- the environment (e.g., seasonal diseases, natural events).

**Fig 5:**
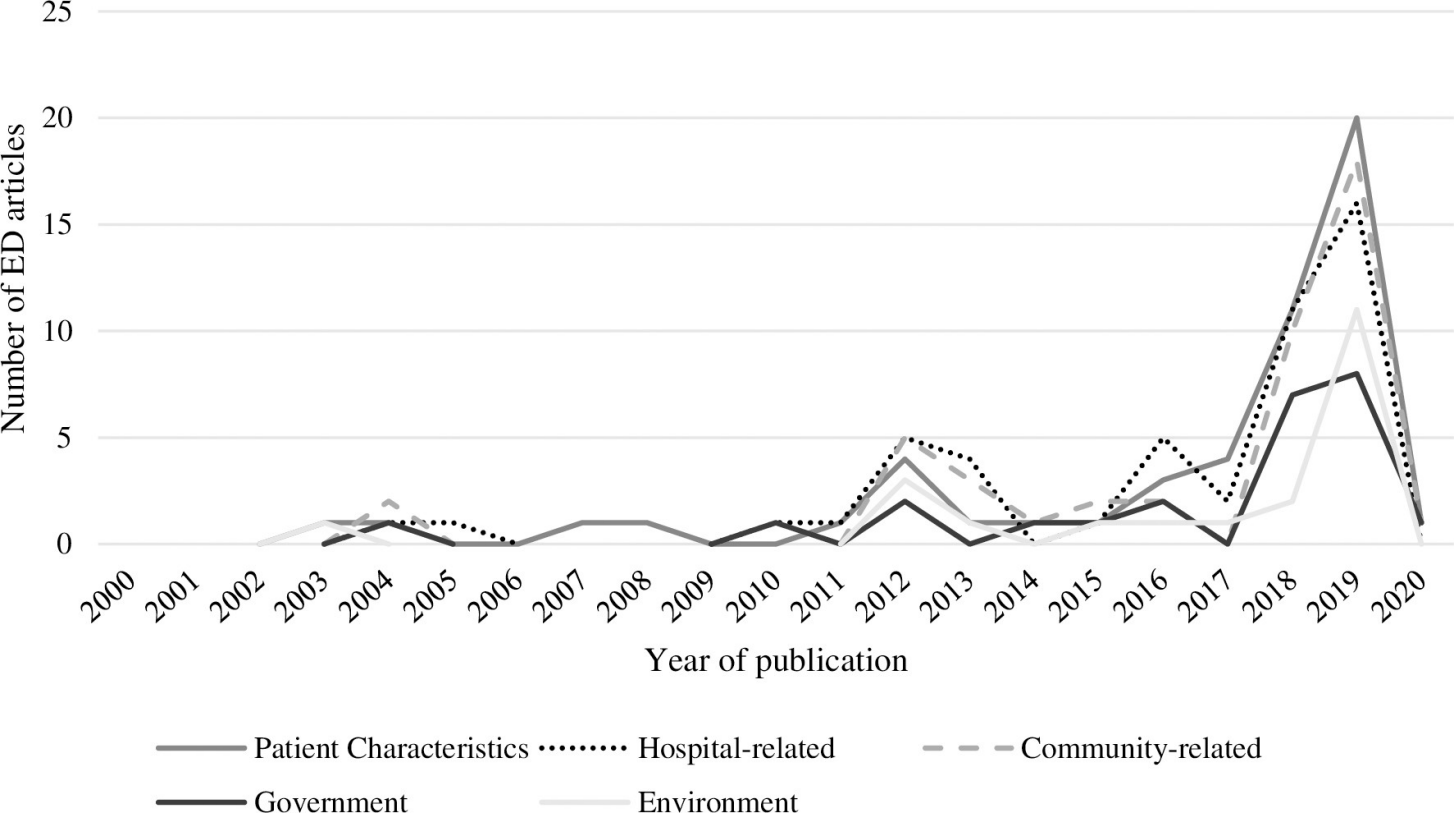
The number of ED articles mentioning causes by type from 2000–2020.

Similar to the reporting of problems, causes were reported in clusters or as a chain of circumstances. For example, ‘drunk’ patients requiring a lot of frontline worker time, as well as the lack of doctors, or increased patient wait time. Fig 5 presents the publication of ED articles mentioning the different causes over the two-decade study period.

Although proposed causes were identified across the full study period, they were more frequently mentioned over the last two years (i.e., 2018 and 2019). Journalists often framed ED problems stemming from different system levels, such as the hospital, the community, or the health system as a whole, e.g., “*problems are being caused by workforce shortages*, *a lack of accessibility to GPs and the state’s ageing population*” [48]. Across the 20 years, but primarily in the last two years, journalists tended to provide an extensive description of patient characteristics and behaviours associated with service use as causing increases in ED service demands: “*DRUNKS are flooding hospital emergency departments taking up beds*, *hogging staff time*” [49], “*clogging hospital emergency departments after hours with innocuous ailments such as hay fever*, *ankle sprains*, *sore backs and ingrown toenails*.” [50], and “*emergency departments are clogged by people with conditions that should be treated by a GP or pharmacist*” [51]. The depiction of environmental events due to the season and national holidays was minimally described across the two decades, being proposed as a cause primarily in 2019: “*The number of notified flu cases this year has hit 1777 compared to 757 at the same time last year*” [52], “*the busy festive season and partying contributed to the admissions*” [53], “*winter was always the busiest period for hospitals*” [54], and “*The unprecedented high rate of ‘summer flu’ was partly responsible*” [55].

### Frame 3: Solutions can improve ED care delivery, but few are implemented

Solutions were presented in 4 different ways: (1) calls for solutions or fixes and recommendations; (2) solutions that had been enacted and were successful; (3) solutions that were inadequate, unsuccessful, or delayed; and (4) interim measures (i.e., temporary solutions). Similar to the representation of problems, solutions were proposed or discussed at different organisational levels of the health system: ward, hospital, community, and government. These are presented in Table 1 below.

**Table 1:** Examples of solutions at the different organizational levels.

	ED Ward	Hospital	Community	Government
**Calls for solutions/ recommendations**	Culturally appropriate space/ waiting room for Indigenous patients; artificial intelligence clinical decision support programs	Open/ reopen beds in wards, 24/7 mental health drop-in centres, efficient discharging processes, mandated nurse-to-patient ratios in the hospital; extra staff	Mobile surgery units for rural areas, better/more community mental health and GP services	Increase in the Medicare rebate for GPs, funding for services, a mandatory reporting of 12+ hour length of stay in ED
**Enacted/ successful**	ED waiting room nurse; colour-coding uniforms and pathology bags; rapid offload policy, rapid transfer nurse position funding, redesigning care program	Separate areas for Indigenous patients to meet/ rest/ engage with specialist hospital staff, Indigenous Liaison officers; flow modelling technology; patient admission and prediction tool	ANU Medical School local training program, vaccination programs	Investment in medical services, reporting to the health minister ED stays of 24+ hours, 4-hour treatment targets
**Inadequate/ failed/ delayed**	CDUs; rapid offload policy	Opening forensic mental health beds, EMR rollout	Public education	New hospital beds promised in the last election campaign; bans to hospital bypass; four-hour treatment targets; funding
**Interim measures**	Send patients to nearby hospitals, treat patients on temporary trollies, roster additional staff, organise additional treatment areas	Opening up a small number of beds; medical assessment units, reopening units for overflow; cancelling less-urgent surgery	Show problem venues graphic photos of their patrons who end up in hospital; home hospital services	Corporate liquidators to run hospitals,

Note. ANU, Australian National University; CDU, Clinical Decision Unit; ED, Emergency Department; EMR, Electronic Medical Record; GP, General Practitioner

### Frame 4: ED systems are difficult to praise as they don’t often meet efficiency performance targets

Praise for EDs, while infrequent, was often described in terms of performance perceptions such as achieving targets and waiting times, e.g., “*The Alfred*, *which was one of just six Victorian hospitals to meet the federal target to treat 75 percent of emergency patients within four hours this year*” [56] and “*That is a slight improvement on the average 43 minute waiting time recorded in 2010–2011*, *when 55 percent patients were seen on time*” [57]. Journalists acknowledged the role of frontline staff in the EDs’ capacity to cope with service demands, e.g., “*Coping with additional demand should be seen as a credit to hardworking medical staff*” [58]. The restructuring of services such as new models of care and changes in funding were also subjects of praise, e.g., “*the hospital appointed an ED waiting room nurse in October to help streamline patient flow*” [59], “*So (there’s) ongoing improvement within the emergency department*, *with more beds to be funded by the Government in the coming 12 months as part of a major refurbishment of the ED*” [60] and “*An extra $45*.*7 million over the next four years has been committed to support the expansion of ED service*” [61].

### Frame 5: The opinions and assertions of patients are undervalued compared with bureaucrats

**Fig 6:**
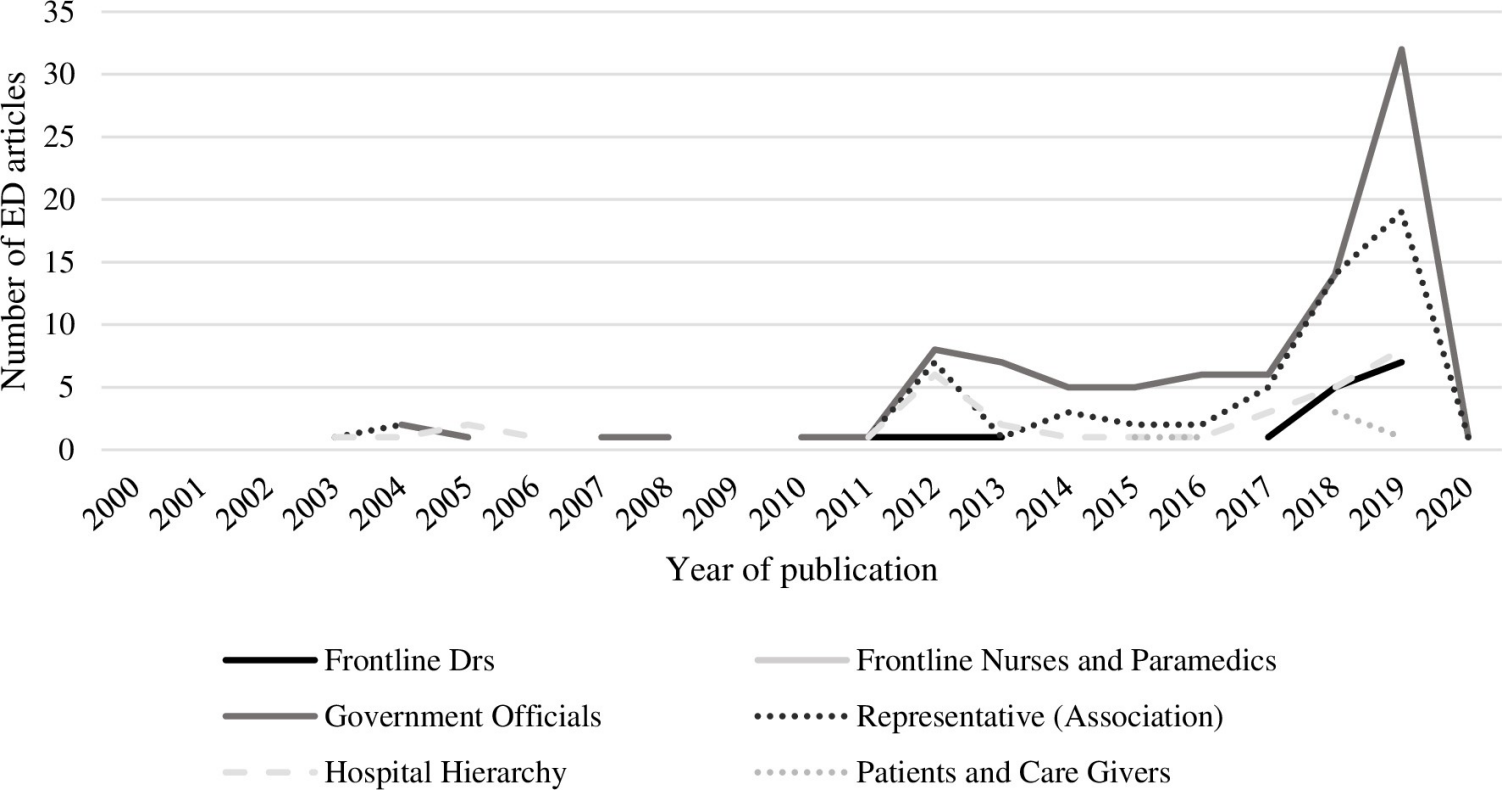
The number of ED articles presenting opinions by voice from 2000–2020.

Journalists tended to provide extensive space for the opinions of state and federal government officials, representative or association representative spokespeople (e.g., Australasian College of Emergency Medicine, ACEM; Australian Medical Association, AMA), and hospital executives on EDs’ performance. Opinions of frontline staff about EDs were also reported. More often than not, doctors’ opinions were reported and referred to by their name or their occupation (e.g., “*Intensive care doctor*”, “*one doctor*”, “*rural doctors*”). However, the opinions of nurses and other frontline staff were minimally reported or absent from ED articles. Similarly, the voice of patients and caregivers were minimally reported or absent from ED articles. Fig 6 represents the number of ED articles that provided space for each voice.

### Frame 6: Sources of information about the ED can increase the perception of poor ED performance

ED performance was often reported as fact with no reference to the source of the information. When sources were cited, journalists referred to information from publicly available government publications, e.g., “*The latest Bureau of Health Information quarterly report*” [55], “*The Productivity Commission’s Report*” [60], “*The 2018–2019 budget*” [55]. Published research and publications from representative bodies (e.g., ACEM, AMA) were also used as sources of information about EDs, e.g., “*new research on emergency waiting lists*, *published in today’s Medical Journal of Australia*” [62], “*The Australasian College for Emergency Medicine (ACEM) data shows “unacceptable access block” and overcrowding with patients in South Australia*, *Tasmania and Canberra most at risk*.” [63], “*The AMA report does show*, *however*, *that the state’s hospitals still failed to meet their elective surgery targets of treating some patients within clinically recommended times and the state faces a cut in federal funding*.” [64]. Other sources of information cited included “*internal documents*” [46], “*leaked reports*” [65], “*incident reports—obtained by ABC News through a freedom of information application*” [46] as well as “*data recorded by the Queensland Ambulance Service*” [66] and “*Figures from Flinders Medical Centre*” [67].

### Discursive strategies

Headlines were typically short and played on themes of capacity (e.g., “*Crisis as ED at double capacity*”; [68], illness (e.g., “*Flu just a sick excuse*” [69]), challenge (e.g., “*ED hospital pains as more doctors needed*”; [70]) and urgency (e.g., “*Emergency Rooms now at crisis point*”; [71]), as well as shaming (e.g., “*Worst-performing emergency departments named*”; [72]). By-lines did not always include author information (86% included author name), while only 30% included author and specialisation title information e.g., “*medical reporter*” and “*health reporter*”. Rhetorical devices such as hyperbole and imagery were used to emphasise problems, e.g., “*chaotic scenes and blown-out patient wait times as emergency department workers scrambled*” [45], the causes of the problems e.g., “*The flood of sick and injured people severely affected wait times across NSW*” [73], and solutions e.g., “*Hospital managers have used the administrative equivalent of drain cleaner to unclog their emergency departments*” [74].

## Discussion

The current study systematically identified and reviewed sampled news stories about public hospital based EDs in Australia, using media frame analysis to examine how the Australian media have portrayed public hospital EDs over two decades from 2000–2020. News media coverage of EDs increased dramatically from 2012, coinciding with the launch of the MyHosptials website in 2011 and the publication of mandated public reporting of public hospital performance data. Data from these websites tell us that the number of people accessing EDs for care has increased over the past two decades (See Fig 2), the overall proportion of patients ‘seen on time’ (i.e., according to health policy defined time frames within which patients should be seen by a doctor for their allocated triage category) has increased since 2014–16, but fewer ED visits are completed within 4 hours than in 2015–2016 [75]. Along with the launch of the MyHosptials website, it is also possible that technology has made it easier for news outlets to re-produce articles with similar themes and similar ’reliable’ spokespeople for comment. Overall, six frames were identified to describe ways in which the media have portrayed a highly biased reality of Australian EDs in newsprint: the ED system is full of problems; the causes of problems are varied and interdependent; solutions can improve ED care delivery, but few are implemented; ED systems are difficult to praise as they don’t often meet efficiency performance targets; the opinions and assertions of patients are undervalued compared with bureaucrats; and sources of information about the ED can increase the perception of poor ED performance.

Media coverage was concerned primarily with reporting problems with and within ED performance and assigning causes for the problems. By prominent and consistent depiction of problems reported in the news media, journalists construct a pseudo-reality that EDs are not able to provide safe, urgent medical care to their community (i.e., perform their functional purpose). Contrary to media reports, reliable and publicly available data indicate that most (74%) patients are ‘seen on time’ and almost all (90%) are seen within 1 hour and 32 minutes [75]. Overall, 69% of ED visits are completed within 4 hours, and most (61%) patients leave the ED after being treated with nearly one-third (31%) being admitted to the hospital for further care [75]. Consistent with the construction of a poorly performing system, journalists add to the narrative that specific patient groups (i.e., predominantly mental illness, alcohol and drug use, non-urgent queries) and limited resources (e.g., workforce, beds) are causing the problems EDs experience. While these patient groups may not present with the most common principal diagnosis, they are likely to spend the longest in the ED, which leads to the perception that they use the majority of resources and drive resource constraints [76, 77]. Coupled together, as they often are in ED news articles, these frames construct a perception of a system that has trouble meeting the needs of a diverse community, in an equitable and timely manner. Many innovations in care delivery (i.e., solutions) have been implemented in EDs, but these are not reported by the news print media [78]. When reported, the way solutions were framed amplified their inadequacy for surmounting the scale of the problem–temporary, or only proposed and not yet implemented. Critically, the analysis magnifies the lack of fit-for-purpose, go-to supports available for these patient groups and the subsequent impact their crisis care needs have on demand and resources in the ED. Consistent with the narrative for problems and causes, the interpretation of solutions emphasises the underdevelopment and lack of detail provided to effectively improve ED systems.

By focusing on the meeting of performance targets, journalists make this the value through which success is determined. Even more troubling, is the biased construction of reality through their ‘interpretation’ of performance targets by determining what is good, what is ok, and what is bad performance. Journalists used information from publicly available government publications to construct a picture of ED performance. The construction of an ‘EDs in crisis’ narrative is part of a general trend towards reporting ‘bad news’ motivated to increase ‘click’ rates, however, focusing on a performance narrative removes the ED context and human aspect of care. The reporting of opinions from doctors and government officials were used to foreground the experience of reality at the frontline and the government’s role in the causes of the problems and the solutions. Doctors and their representative organisations (e.g., AMA, ACEM) leverage the ‘bad press’ to advocate for increased funding and structural changes in ED to support better care. However, the limited number of perspectives, particularly the absence of the patient, family members, and non-medical healthcare professional voices, leads to a highly biased construction of reality. For example, while clinicians perceive that prompt care is paramount, patients and family members value communication and active participation in their ED experience [79]. Compounding the limited sourcing of perspectives, journalists frequently reported information as fact with no reference to the source, limiting readers’ ability to independently evaluate the information. Finally, short catchy, and sensationalised headlines that play on ED functions, such as capacity, illness, challenge, and urgency were used to prime readers and draw attention to dominant narratives of problems and causes of problems.

### Limited perspectives

The increase in reporting on EDs, as we see in the data around 2019, could have created news reporting conditions that lean towards the use of ‘limited perspectives’, increasing the potential for misinformation to be reported. The online format, where articles are written with more limited space, may facilitate framing a more argumentative, finger-pointing narrative, one that reflects bureaucratic rather than patient voices. Restricting the diversity of perspectives (e.g., not including patients) perpetuates a paternalistic view of medicine–the only people with authority to speak on medical issues are the doctors and politicians, and patients are seen as unreliable narrators in their own medical stories. Consequently, the public misses out on the opportunity to relate to their peers, regular people experiencing medical issues in their everyday lives.

The general public learns a lot about health care services and health conditions from the news media. The attention news media give to processes and experiences influences public behaviour. For example, increases in news media stories regarding infectious diseases are associated with an increase in testing for the disease [80, 81]. Similarly, public health television advertising campaigns conveying the warning signs of stroke coincided with an increase in the number of ED visits for stroke during the campaign period [82], and the spin in health news stories affected patients’ and caregivers’ interpretation of pharmacological treatments [2]. Consistent with existing literature, the current study found EDs to be portrayed in an unconstructive way with limited positive reporting of successes or achievements. This is in spite of the challenges faced by health care professionals working with diverse populations, in this high-demand and responsive healthcare area [22]. Thus, public willingness to access health care services, such as EDs, may be influenced by the news framing of poor ED functioning.

The way EDs function is influenced by external and internal factors, which have been reported on more frequently over the last couple of years, suggesting a shift in focus on identifying the cause, rather than reporting on the event only. Journalists depend on sources for information and the focus of journalistic attention has implications for sources [29]. For example, journalists’ coverage of medical errors based on reports and interviews with doctors can lead to changes in legislation, policy, and practice [29]. Similarly, journalists’ use of publicly available criminal testimony and court proceedings skewed the construction of mental health reality as criminal and violent [12]. However, there continues to be limited journalist engagement with people using EDs and non-medical health care professionals working in EDs, which repeatedly deprives them of a voice. This restricts contextual information limiting full comprehension of the situation, which can ultimately present a biased view to the public. Large organisations such as AMA and ACEM frequently provide comment for articles about EDs, however, nurses remain invisible in the news media, despite their valuable role in ED care [83]. Consequently, the public misses out on the valuable perspectives that nurses can provide on issues surrounding the ED [83].

### Limitations

The analysis was restricted to news coverage of EDs in Australian newspapers, excluding coverage of specific events and infectious diseases (e.g., COVID-19). Expanding the analysis to the coverage of specific events and infectious diseases, as well as to additional sources of media coverage, will add to the existing knowledge on the portrayal of EDs in the news. Over the last 20 years (i.e., 2000–2020), there has been increasing news media attention given to EDs. This increase coincides with greater access to public reporting of public hospital data. In particular, news reporting of problems has increased over the last decade. However, it is not clear if the number of problems has increased or if the increase in reporting is due to the accessibility of hospital performance data. This can be an interesting line of inquiry if pursued in future studies. Finally, the analysis excluded articles reporting a specific event, case or communicable disease. This may have biased results by systematically excluding ‘good news stories’ (individual positive cases) or positive (or negative) framing of ED response to challenging events (e.g., thunderstorm asthma, mass-casualty events).

## Conclusions

As in the film Groundhog Day [84], news media reporting on EDs in Australia is stuck in a time loop, reporting the same themes over and over again. The strong impression conveyed by these news stories is that EDs are not functioning due to the many problems that they experience, and that those problems are caused by under-resourcing (i.e., funding, staff, hospital infrastructure), inappropriate use by patients, and increasing population. By framing stories on EDs and healthcare in this way, the news media are likely to exert a powerful influence on public responses to health service problems. Importantly, they may lead people to avoid attending EDs when necessary and be dissatisfied with the support available to them when in crisis. As populations increase and chronic conditions become more complex with comorbidities becoming the norm, EDs will become more and more integrated into continuous care. Given this, it is important to investigate how ED stories presented in the news media selectively present facts, themes, and claims, thereby possibly limiting the true understanding of a functioning health service.

## Supporting information

S1 Checklist PRISMA 2020 checklist. (DOCX)

S1 Table Complete list of included articles. (DOCX)

S1 Appendix (DOCX)
