# Supplementary material for: Groundhog Day in the emergency department: A systematic review of 20 years of news coverage in Australia
Source: PLoS One. 2023 May 2;18(5):e0285207. doi: 10.1371/journal.pone.0285207 (PMC10153716; doi:10.1371/journal.pone.0285207)
Supplement: S1 Table — (DOCX) [file pone.0285207.s002.docx]

Table S1. Complete list of included articles.

| **Newspaper** | **Date of publication** | **Title** | **Authors** |
| --- | --- | --- | --- |
| Adelaide Advertiser | 6/08/2003 | Elderly patients putting added strain on bed shortage hospitals' winter of discontent | Barry Hailstone |
| Courier Mail | 30/08/2003 | After-hours services go from sad to sick | Leanne Edmiston |
| Australian Broadcasting Corporation | 20/10/2003 | Minister applauds Rockhampton hospital waiting times | Unknown |
| Courier Mail | 19/11/2003 | Five days in emergency unit end in transfer | Hedley Thomas |
| Adelaide Advertiser | 1/12/2003 | extra beds at flinders | Jill Pengelley |
| Courier Mail | 19/01/2004 | Emergency funding needed for new beds | Leanne Edmiston |
| The Advertiser | 21/02/2004 | Overload emergency patient admissions jump by 12,000 | Melissa King and Laura Anderson |
| The Australian | 29/04/2004 | Hospital's assembly-line cure-all - diagnosis critical: Inside our hospital system | Jeremy Roberts |
| The Advertiser | 12/05/2004 | FMC death rate inquiry | Unknown |
| The Advertiser | 28/06/2004 | More hospital beds but emergency care concern | Laura Anderson |
| The West Australian | 5/01/2005 | Hospital queues getting longer | Katherine Fleming |
| The West Australian | 8/01/2005 | Hospital makes it faster for patients | Katherine Fleming |
| The Advertiser | 28/01/2005 | More surgery, less waiting at flinders | Rebecca Jenkins |
| The West Australian | 30/03/2006 | Suicidal man 'should have been admitted' | Roy Gibson |
| The Australian | 30/10/2006 | Triage rethink cuts hospital wait times | Clara Pirani |
| Australian Broadcasting Corporation | 21/11/2006 | Wait for beds at tweed heads | Unknown |
| The West Australian | 23/01/2007 | Doctors fear long weekend disaster | Cathy O'leary |
| The West Australian | 23/03/2007 | Emergency patients wait all day for a bed | Cathy O'leary |
| Australian Broadcasting Corporation | 7/05/2007 | Emergency dept numbers on the rise | Unknown |
| The Advertiser | 28/05/2007 | Emergency patients at RAH left to wait | Jill Pengelley |
| The West Australian | 2/08/2007 | Acute bed crisis worst in WA | Cathy O'leary |
| The West Australian | 22/09/2007 | Hospital targets relaxed: AMA | Cathy O'leary |
| The Australian | 23/10/2007 | Lance bloated beast of bureaucracy | Malcolm Colless |
| Australian Broadcasting Corporation | 25/10/2007 | Corbell defends hospital system after damning report | Unknown |
| Australian Broadcasting Corporation | 25/10/2007 | AMA releases damning report on public hospitals states savaged over hospitals record | Unknown |
| Sydney Morning Herald | 26/10/2007 | Crowded hospitals in danger zone | Natasha Wallace |
| The West Australian | 10/04/2008 | Ambulance ramping fuels winter concerns | Cathy O'leary |
| Herald Sun | 1/07/2008 | Hospitals barely cope longest wait for surgery, emergency care | Ben Packham |
| The West Australian | 5/09/2008 | Hospital beds, staff still key issues eight years on | Unknown |
| The West Australian | 10/09/2008 | Dying risk 'up 30pc in crowded hospitals' | Cathy O'leary |
| The West Australian | 20/09/2008 | Plea for action on hospital overcrowding | Cathy O'leary |
| Courier Mail | 11/10/2008 | Eight-hour wait for patients | Janelle Miles |
| The Advertiser | 13/11/2008 | needless' hospital deaths | Tory Shepherd |
| The West Australian | 31/01/2009 | Hospitals fare poorly again on response times | Cathy O'leary |
| Herald Sun | 6/04/2009 | Crowds can be a killer | Janelle Miles |
| The West Australian | 23/06/2009 | Hospitals battle on four-hour rule | Cathy O’leary |
| The Australian | 30/06/2009 | Hospitals still lag in treatment time | Sid Maher |
| Courier Mail | 30/07/2009 | Flu crisis cuts ops - pandemic takes over hospitals | Rosemary Odgers |
| Herald Sun | 14/08/2009 | As the PM inspects Victoria's hospitals, one senior doctor pleads ... Fix our ailing system | Unknown |
| Australian Broadcasting Corporation | 8/01/2010 | Coffs Harbour hospital Emergency Department at crisis point | Unknown |
| Daily Telegraph | 10/02/2010 | after 8 hours in emergency, 27% of patients won’t have a wards bed -- situation critical | Steve Lewis |
| The West Australian | 25/05/2011 | Brazen drinking culture to blame | Unknown |
| Australian Broadcasting Corporation | 6/10/2011 | Emergency doctor: we can't cope Hospital 'overcrowded, overwhelmed' EXCLUSIVE | Julia Medew |
| The Age | 18/10/2011 | The state of emergency | Lesley Russell |
| Australian Broadcasting Corporation | 5/01/2012 | Increase in emergency department admissions | Unknown |
| The Australian | 6/02/2012 | Emergency treatment target 'saving lives' | Adam Cresswell |
| The Age | 6/02/2012 | Researchers back emergency ward four hour rule | Kate Hagen |
| Courier Mail | 7/03/2012 | Emergency cases now waiting twice as long | Peter Hall |
| Australian Broadcasting Corporation | 12/06/2012 | Emergency dept feeling GP shortage | Unknown |
| Australian Broadcasting Corporation | 30/07/2012 | Rally for Bulli Hospital ED attracts 500 | Unknown |
| Australian Broadcasting Corporation | 13/08/2012 | Doctors call for more beds at the Mater | Unknown |
| Courier Mail | 6/09/2012 | Vital signs poor for GP home visits | Stuart Tait |
| Australian Broadcasting Corporation | 6/09/2012 | Unseasonal urgent patient spike at Wollongong Hospital's ED | Unknown |
| Courier Mail | 13/09/2012 | State waiting times improve | Sue Dunlevy |
| The Age | 28/09/2012 | Funding for Victorian hospitals at risk | Kate Hagen |
| Australian Broadcasting Corporation | 28/09/2012 | 'Timeliness' still a problem in ACT emergency dept | Kathleen Dyett |
| Australian Broadcasting Corporation | 12/12/2012 | Hospital says ED care up to speed | Unknown |
| Sydney Morning Herald | 14/12/2012 | Emergency: NSW dominates list of worst hospitals | Amy Coderoy |
| Daily Telegraph | 14/12/2012 | Day's wait to get a bed Patients held in emergency | Evelyn Yamine |
| Courier Mail | 14/12/2012 | Delays clog up hospital system | Janelle Miles |
| **Australian Broadcasting Corporation** | **4/01/2013** | **A busy New Year's Day for ED** | **Unknown** |
| Courier Mail | 8/01/2013 | Bypass farce - access delays continue at busy hospital | Peter Hall |
| Courier Mail | 27/02/2013 | Reduction in patient wait-times | Steven Wardill |
| The Australian | 28/02/2013 | Patients shackled in hospital corridors | Michael Owen |
| The Australian | 1/03/2013 | Shackles 'a regrettable reality' | Michael Owen |
| The Australian | 23/04/2013 | Flow modelling cuts emergency wait times | Jennifer Foreshew |
| The Age | 26/07/2013 | Frankston hospital failing time targets | Kate Hagen |
| The Advertiser | 26/07/2013 | Emergency wards failure | Brad Crouch |
| Courier Mail | 26/07/2013 | Hospitals in recovery | Sue Dunlevy |
| Australian Broadcasting Corporation | 26/07/2013 | John Hunter ED fails treatment benchmark | Unknown |
| The Advertiser | 18/10/2013 | Emergency wait leads nation | Jordanna Schriever |
| Courier Mail | 18/10/2013 | Patients choosing hospital over GPs | Brittany Vonow |
| Courier Mail | 14/02/2014 | Surgery wait best in nation | Sue Dunlevy |
| Courier Mail | 10/03/2014 | Prepared for emergency | Kelmeny Fraser |
| Courier Mail | 9/04/2014 | Co-payments not to be sneezed at | Simon Benson |
| Australian Broadcasting Corporation | 15/04/2014 | Home visit GP service aims to ease pressure on hospital emergency departments | Unknown |
| Australian Broadcasting Corporation | 30/04/2014 | Canberra hospitals record worst emergency department wait times | Unknown |
| The Age | 28/05/2014 | Footscray, Frankston among slowest-performing emergency departments | Kate Hagen |
| The Age | 29/05/2014 | Worst-performing emergency departments named | Kate Hagen |
| Sydney Morning Herald | 24/06/2014 | Fast data ramps up ambulance's hospital service | Trevor Clarke |
| The Advertiser | 29/08/2014 | Treat 'richer before poorer' | Sue Dunlevy |
| Australian Broadcasting Corporation | 5/09/2014 | Hunter Health acknowledge waiting times at John Hunter Hospital's ED can be further improved | Unknown |
| Daily Telegraph | 11/09/2014 | Hold the phone on the emergency department | Jason Morrison |
| Courier Mail | 27/09/2014 | Drunks clogging up public hospitals | Sarah Vogler |
| Sydney Morning Herald | 1/11/2014 | Long hours spent lying on hospital trolley reflect poorly on our healthcare | Harold Mitchell |
| Sydney Morning Herald | 9/01/2015 | Emergency wait targets hidden by short stay units | Harriet Alexander |
| The Australian | 13/01/2015 | Waiting time targets in doubt | Mark Coultan |
| **Australian Broadcasting Corporation** | **4/02/2015** | **ACT among Australia's worst for public hospital waiting times, satisfaction levels: report** | **Lisa Mosley** |
| Australian Broadcasting Corporation | 4/03/2015 | Hunter hospital EDs fall below target for wait times | Unknown |
| Australian Broadcasting Corporation | 4/03/2015 | Victorian hospitals: Number of people using emergency departments jumps 53pc, report shows | Peter Lusted |
| Australian Broadcasting Corporation | 14/05/2015 | Public hospital patients give Hunter EDs tick of approval | Unknown |
| The Advertiser | 16/06/2015 | Code white - hospitals join the critical list | Brad Crouch |
| The Advertiser | 7/11/2015 | Doctor's ramping warning | Brad Crouch |
| Courier Mail | 4/12/2015 | Mental health bed shortage in SA | Unkown |
| Australian Broadcasting Corporation | 28/01/2016 | Queensland public hospitals fail to meet national targets in Australian Medical Association report card | Andree Withey and Nick Wiggins |
| The West Australian | 24/05/2016 | Lives at risk from long ED waiting times | Cathy O’leary |
| Sydney Morning Herald | 2/06/2016 | 'Trolley block' in emergency departments on the decline | Harriet Alexander |
| The Advertiser | 3/06/2016 | Patient waits four days for RAH bed | Brad Crouch |
| The Advertiser | 7/06/2016 | ED gridlock eases for less than a day - then overload continues | Brad Crouch |
| Courier Mail | 28/06/2016 | Sooks are health hazard | Rose Brennan |
| Australian Broadcasting Corporation | 21/07/2016 | Royal Hobart Hospital staff urged to speed up patient turnover to ease bed pressure in emergency | Pablo Vinales |
| **The Age** | **3/08/2016** | **A growing emergency inside our hospitals** | **Unknown** |
| Courier Mail | 22/08/2016 | 3 AM strikes - your out | Sam Phillips |
| The Advertiser | 23/08/2016 | Ramping, 24 hours for bed at hospitals | Katrina Stokes |
| Australian Broadcasting Corporation | 4/09/2016 | Labor calls for urgent care clinics to take pressure off Western Australian emergency departments | Rebecca Carmody and Briana Shepherd |
| Courier Mail | 10/09/2016 | ED delays put lives in danger | Charlie Peel |
| The West Australian | 20/09/2016 | It’s no mystery why patients turn to EDs | Cathy O'leary |
| The West Australian | 3/11/2016 | Ambos’ mobile blood work helps EDs | Cathy O'leary |
| The Advertiser | 9/12/2016 | Doctors on call for 48 hours | Erin Jones |
| Sydney Morning Herald | 17/01/2017 | Sydney hospital forced to close to ambulances | Kate Aubusson |
| The Advertiser | 23/01/2017 | Boozy price of Australia day | Josephine Lim |
| Australian Broadcasting Corporation | 17/02/2017 | AMA public hospital report shows 'woeful' and underfunded system | Lee Brooks and Clare Blumer |
| The Age | 18/02/2017 | Hospitals in 'emergency' state | Kate Aubusson and Rania Spooner |
| Sydney Morning Herald | 18/02/2017 | Hospitals 'in constant state of emergency' | Kate Aubusson |
| Herald Sun | 19/02/2017 | BEDS FOR BOOZERS | Kathryn Powley |
| The West Australian | 23/02/2017 | Emergency plea | Cathy O'leary |
| Australian Broadcasting Corporation | 28/02/2017 | Hospital wait outrage leads to more beds, questions over 'culture of secrecy' | Richard Baines and James Dunlevie |
| Sydney Morning Herald | 15/03/2017 | Hospital staff confront a tidal wave of patients | Kate Aubusson |
| The Australian | 29/05/2017 | Hospital wait times ‘sanitised’ | Unknown |
| Herald Sun | 10/06/2017 | Rebate cut ‘care crisis’ | Sue Dunlevy |
| Sydney Morning Herald | 27/06/2017 | Clock ticking for after-hours doctors | Adam Gartrell |
| Australian Broadcasting Corporation | 30/06/2017 | Patient describes 'horrible' wait for mental health bed in Royal Hobart Hospital | Linda Hunt |
| Australian Broadcasting Corporation | 15/08/2017 | WA hospital emergency departments under fresh strain; GPs examine overcrowding | Michaela Carr |
| The Australian | 18/08/2017 | Free flu jab could save us millions | Sue Neales |
| The Age | 26/08/2017 | Ambulances queue outside hospitals as flu cases surge | Liam Mannix and Benjamin Preiss |
| Australian Broadcasting Corporation | 18/09/2017 | Royal Hobart Hospital overloaded, staff under 'intense pressure', union says | Emilie Gramenz |
| The Advertiser | 28/09/2017 | Hospitals battle new patient ills | Brad Crouch |
| The Advertiser | 2/12/2017 | MY RAH HELL | Brad Crouch |
| Daily Telegraph | 30/01/2018 | Testing our patients | Rose Brennan |
| Australian Broadcasting Corporation | 6/02/2018 | Royal Darwin Hospital overcrowding at crisis point, significant underfunding, AMA says | Unknown |
| Courier Mail | 7/02/2018 | Emergency call to not clog casualty | Cameron Domanii |
| Australian Broadcasting Corporation | 7/02/2018 | NT Government concerned for Royal Darwin Hospital life span as 'code yellow' cancelled | Unknown |
| The Age | 26/02/2018 | Emergency stays harm mentally ill | Aisha Dow |
| Sydney Morning Herald | 26/02/2018 | Hospitals fail mental health patients | Aisha Dow |
| Courier Mail | 27/02/2018 | Don’t be a pain the ERs | Steven Scott |
| Australian Broadcasting Corporation | 1/03/2018 | Doctor warns Tasmania's 'health crisis' will cost lives and more funding needed | Rhiana Whitson |
| The Advertiser | 3/03/2018 | Gridlock for RAH ward beds | Brad Crouch |
| The Advertiser | 14/03/2018 | ED doctors warn health system is ‘in crisis’ | Brad Crouch |
| The Advertiser | 29/03/2018 | Bed shortage at RAH close to a ‘disaster’ | Brad Crouch |
| Australian Broadcasting Corporation | 29/03/2018 | Mental health 'crisis' as patients endure long waits at hospital emergency departments | Rebecca Turner |
| Australian Broadcasting Corporation | 2/04/2018 | Royal Hobart Hospital: What is causing the problems at Tasmania's major health facility? | Rhiannon Shine |
| The Australian | 4/04/2018 | Call to lift indigenous medical staff numbers | Sam Buckingham-Jones |
| Courier Mail | 7/04/2018 | Hospital pass | Des Houghton |
| Courier Mail | 13/04/2018 | Bed shortage hits sick kids | Des Houghton |
| The Advertiser | 5/05/2018 | Patient shackled to bed, bottle used for a toilet | Katrina Stokes |
| Australian Broadcasting Corporation | 8/05/2018 | Physical restraint of patients with drug-induced psychosis needs rethink, health researcher says | Patrick Martin |
| Courier Mail | 14/05/2018 | Cutting wait times faces 4-year delay | Steven Scott |
| The Australian | 15/05/2018 | A state of emergency | Michael Owen |
| The Advertiser | 13/07/2018 | Crowded EDs put patients 'at risk' | Brad Crouch |
| The Advertiser | 17/07/2018 | Hospital EDs full — yet again | Brad Crouch |
| The West Australian | 21/07/2018 | Eight-hour ED wait for mentally ill | Rourke Walsh and Gabrielle Knowles |
| Courier Mail | 8/08/2018 | Hospitals have lost patients | Talisa Eley |
| Courier Mail | 10/08/2018 | One in four emergency visits trivial | Madura Mccormack |
| The Advertiser | 4/10/2018 | Crisis as ED at double capacity | Elizabeth Henson |
| Australian Broadcasting Corporation | 8/10/2018 | Emergency departments in 'crisis' as mental health patients left waiting: new report | Olivia Willis |
| The Advertiser | 7/11/2018 | Record hospital ramping | Brad Crouch |
| The Australian | 6/12/2018 | Hospital wait times grow by two days a year | Sean Parnell |
| The Advertiser | 6/12/2018 | Our hospital EDs worst performers in Australia | Brad Crouch |
| Sydney Morning Herald | 12/12/2018 | Emergency wait times blow out at major Sydney hospital | Kate Aubusson |
| Herald Sun | 12/12/2018 | Booze battlegrounds | Grant Mcarthure |
| Courier Mail | 10/01/2019 | Staff lose patience over ramping crisis | Jill Poulsen |
| Courier Mail | 27/01/2019 | The doctor can't see you | Greg Stolz |
| The Age | 30/01/2019 | Mentally ill left waiting in hospitals | Dana Mccauley |
| Courier Mail | 3/02/2019 | Hospitals nail delay problems | Natasha Bita |
| Courier Mail | 8/02/2019 | Doctor deserves praise for warning on hospital | Unknown |
| Courier Mail | 9/02/2019 | Pop-up medical ward | Janelle Miles |
| The Advertiser | 21/02/2019 | Calls ramp up to open more beds at Flinders | Brad Crouch |
| Daily Telegraph | 24/02/2019 | Nightmare waits out west | Jane Hansen |
| Australian Broadcasting Corporation | 27/02/2019 | Documents reveal the harm ramping is causing Royal Adelaide Hospital patients | Claire Campbell |
| Courier Mail | 6/03/2019 | Bid to boost hospital’s ED | Erin Smith |
| Australian Broadcasting Corporation | 12/03/2019 | Sir Charles Gairdner Hospital emergency chief warns of 'avoidable deaths' due to staff shortages | Nicolas Perpitch |
| The Australian | 18/03/2019 | Shorten to pledge $1bn for hospital upgrades | Joe Kelly |
| Courier Mail | 28/03/2019 | Hospitals on life support | Domanii Cameron and Janelle Miles |
| Courier Mail | 28/03/2019 | Far north affected by capacity nightmare | Peter Michael |
| The Advertiser | 1/04/2019 | Plans to give EDs 12 hour deadline to admit patients to wards | Elizabeth Henson |
| Courier Mail | 3/04/2019 | Epidemic fuelling our hospital crisis | Janelle Miles |
| The Australian | 4/04/2019 | Mentally ill clog crisis-care beds | Luke Griffiths |
| The Australian | 10/04/2019 | Adelaide's emergency waiting times 'as bad as it gets' | Luke Griffiths |
| The Advertiser | 10/04/2019 | ED hospital pains as more doctors needed | Brad Crouch |
| Courier Mail | 10/04/2019 | Paramedics ‘kicked out’ by ED staff | Jessica Marszalek |
| Courier Mail | 11/04/2019 | Ambos, nurses at war | Jessica Marszalek |
| Courier Mail | 11/04/2019 | Emergency Rooms now at crisis point | Jessica Marszalek |
| Courier Mail | 11/04/2019 | Sick suffer in health turf war | Jessica Marszalek |
| The Advertiser | 18/04/2019 | Health centres to ease strain on winter EDs | Adam Langenberg |
| **Courier Mail** | **19/04/2019** | **400 beds needed to get hospitals off critical list** | **Des Houghton** |
| Courier Mail | 20/04/2019 | Hospitals on critical list | Des Houghton |
| Courier Mail | 4/05/2019 | Hospitals where emergency wait is more than a day | Janelle Miles |
| The Advertiser | 5/05/2019 | Shorten's pledge to fix EDs | Annika Smethurst |
| Herald Sun | 5/05/2019 | Hospital wait vow | Annika Smethurst |
| Courier Mail | 5/05/2019 | Emergency wait shambles | Janelle Miles |
| Courier Mail | 6/05/2019 | QHealth under fire for ‘making excuses’ on wait times | Janelle Miles |
| Courier Mail | 7/05/2019 | I don't want to lose my patients | Janelle Miles |
| Courier Mail | 7/05/2019 | Patients sick of silent treatment | Jessica Marszalek |
| Courier Mail | 9/05/2019 | ‘We were saying this child can’t breathe please help him’ | Andrea Macleod |
| Australian Broadcasting Corporation | 15/05/2019 | Tasmanian Health Service pushes ahead with controversial hospital overcrowding protocol | Rhiana Whitson |
| The Australian | 29/05/2019 | Patients 'not safe' in emergency wards | Matthew Denholm |
| Sydney Morning Herald | 12/06/2019 | Treatment times blow out at overcrowded emergency departments | Kate Aubusson |
| **Sydney Morning Herald** | **12/06/2019** | **3 million patients to cram emergency departments** | **Kate Aubusson** |
| Sydney Morning Herald | 12/06/2019 | We keep pumping money into a system we know is at breaking point | Carmel Tebbutt |
| Daily Telegraph | 15/06/2019 | Ambos sound a siren on safety | Clarissa Bye |
| Herald Sun | 16/06/2019 | Did emergency wait end in baby’s death? | Ed Gardiner |
| The Advertiser | 24/06/2019 | Flu-hit hospitals send emergency message | Lauren Novak |
| Herald Sun | 28/06/2019 | Tis the season of folly | Grant Mcarthure |
| Courier Mail | 2/07/2019 | Crisis ramping up in a sick health system | Michael Wray |
| Courier Mail | 3/07/2019 | Health figures add up to a system in crisis | Jessica Marszalek |
| Courier Mail | 8/07/2019 | Flu just a sick excuse | Cameron Domanii |
| Herald Sun | 22/07/2019 | A sickly health system leads to an ailing nation | Elizabeth Sigston |
| Courier Mail | 8/08/2019 | Hospital in revolt over ‘catastrophic’ ramping | Unknown |
| Sydney Morning Herald | 21/08/2019 | Inhumane' wait times for mental health illness care | Kate Aubusson |
| The Advertiser | 4/09/2019 | Cardiac test to slash ED queues | Brad Crouch |
| Courier Mail | 12/09/2019 | Broken records | Jessica Marszalek |
| Courier Mail | 12/09/2019 | Chaos amid latest hospitals crisis | Unknown |
| Courier Mail | 2/10/2019 | Sick and AMP; Tired of waiting | Jessica Marszalek |
| Courier Mail | 2/10/2019 | Too slow, even with extra staff | Jessica Marszalek |
| Courier Mail | 5/10/2019 | Spike nothing to sneeze at | Janelle Miles |
| The Advertiser | 23/10/2019 | Hospital beds close but EDs full | Brad Crouch |
| Australian Broadcasting Corporation | 30/10/2019 | More suicide attempts in RHH emergency department, director tells inquest | Phoebe Hosier |
| Australian Broadcasting Corporation | 31/10/2019 | 1,800 patients waited more than 24 hours in the Royal Hobart Hospital's ED | Phoebe Hosier |
| The Age | 23/11/2019 | Every day on the job is a matter of life and death | Helen Hawkes |
| Courier Mail | 25/11/2019 | It's not right in the ED | Unknown |
| The Australian | 29/11/2019 | Critical care fail to keep up pace | Unknown |
| The Australian | 29/11/2019 | Emergency mental care 'failing patients' | Unknown |
| Sydney Morning Herald | 29/11/2019 | Particles in smoke add to bushfire health risks | Kate Aubusson |
| Courier Mail | 1/12/2019 | Guardian Angels | Unknown |
| Australian Broadcasting Corporation | 5/12/2019 | Adelaide hospital gets $86m injection for beds and staff to ease ramping | Leah Maclennan |
| The Advertiser | 6/12/2019 | Flinders to ease ramping bedlam | Brad Crouch |
| Sydney Morning Herald | 11/12/2019 | Hospitals in 'critical condition': 3 million flood NSW emergency departments | Kate Aubusson |
| Sydney Morning Herald | 13/12/2019 | Public health risk from bushfire smoke a 'wake-up call' | Kate Aubusson |
| Sydney Morning Herald | 16/12/2019 | Urgent action needed to address air quality 'emergency': alliance | Jenny Noyes |
| Sydney Morning Herald | 17/12/2019 | health experts working in 'evidence-free zone' | Kate Aubusson and Nigel Gladstone |
| The Age | 28/12/2019 | Rural doctors call for urgent hospital fix | Melissa Cunningham and Benjamin Preiss |
| The Australian | 8/01/2020 | Hospital System stretched to limit | Unknown |
| The Australian | 8/01/2020 | Higher costs, smarter health | Unknown |
| The Australian | 10/01/2020 | Failing mental and dental health resources driving hospital crisis | Unknown |
| The Australian | 14/01/2020 | Solar safety in the line of fire | Unknown |

Note. Bolded text indicates articles used to identify initial frames and subframes.
