# Supplementary material for: Groundhog Day in the emergency department: A systematic review of 20 years of news coverage in Australia
Source: PLoS One. 2023 May 2;18(5):e0285207. doi: 10.1371/journal.pone.0285207 (PMC10153716; doi:10.1371/journal.pone.0285207)
Supplement: S1 Appendix — (DOCX) [file pone.0285207.s003.docx]

Funding for Victorian hospitals at risk

Hagan, Kate . The Age ; Melbourne, Vic. [Melbourne, Vic]28 Sep 2012: 3.

[
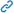
ProQuest document link](http://simsrad.net.ocs.mq.edu.au/login?url=https%3A//search.proquest.com/docview/1080759102%3Faccountid%3D12219)

# ABSTRACT

VICTORIAN hospitals are treating fewer patients in emergency departments within four hours despite new federal targets for them to treat more patients in that time, putting at risk more than...

# FULL TEXT

EMERGENCY PATIENTS TREATED WITHIN FOUR HOURS

As of June 30 Dec 31 Jan 1 result target Victoria 65.9% 65% 72%

NSW 61.8% 60% 69%

QLD 63.8% 64% 70%

WA 71.3% 79% 76%

SA 59.4% 64% 67%

TAS 66% 66% 72%

ACT 55.8% 58% 64%

NT 66.2% 65% 69%

VICTORIAN hospitals are treating fewer patients in emergency departments within four hours despite new federal targets for them to treat more patients in that time, putting at risk more than $12 million in funding this year.

New data released by the Australian Institute of Health and Welfare today shows that just 65 per cent of patients in Victorian emergency departments were treated within four hours in 2011-12, well short of the 72 per cent required under new targets.

Victorian hospitals were treating 65.9 per cent of emergency patients within four hours when the new targets were introduced in January, so they have gone backwards in the first six months of this year.

Doctors say Victorian hospitals are unlikely to reach this year's 72 per cent target, putting Victoria at risk of losing

$12.4 million in funding to be paid to states that meet it.

By 2015, 90 per cent of Australian patients will be required to be admitted to a bed or discharged home within four hours, but the federal government has set interim deadlines that vary between states so they improve on past performance.

Across Australia in 2011-12, 64 per cent of patients were treated within four hours in emergency departments. Western Australia, which introduced the four-hour rule in 2009, had the highest percentage of patients treated within four hours, at 79 per cent, passing its goal of 76 per cent for this year.

New South Wales was among the worst performers, with 60 per cent of emergency patients treated within four hours -- less than the 61.8 per cent of patients treated within that time in January and short of its 69 per cent target for this year.

Australian Medical Association Victorian president Stephen Parnis said improved triage processes and admitting patients to short-stay units for monitoring were "making differences around the margins", but more beds were needed to meet demand from a growing and ageing population.

"The best innovation will only get you so far -- it's an inescapable reality that we need to have the capacity to service the increased demand," he said.

"My feeling is that these targets are unlikely to be met, and it's not from a lack of effort on the part of health services."

The chairman of the Victorian faculty of the Australasian College for Emergency Medicine, Simon Judkins, said patients often waited four hours to be admitted to a ward "even when we can identify as soon as the ambulance turns up that the patient needs a bed".

Changes needed to be made throughout hospitals, not just in emergency departments, he said, including more efficient processes for discharging patients to free up beds.

Victorian Health Minister David Davis said the four-hour rule was unrealistic. "We are concerned that the Commonwealth may withhold valuable funding which will affect all Victorian health services," he said.

"We also note that the Commonwealth government has not expanded GP services, particularly after-hours, to avoid unnecessary ED visits."

Credit: KATE HAGAN HEALTH REPORTER

# DETAILS

**Subject:** Hospitals; Emergency services; Emergency medical care; Health services

**Publication title:** The Age; Melbourne, Vic.

**First page:** 3

**Publication year:** 2012

**Publication date:** Sep 28, 2012

**Publisher:** Fairfax Digital

**Place of publication:** Melbourne, Vic.

**Country of publication:** Australia, Melbourne, Vic.

**Publication subject:** General Interest Periodicals--Australia

**ISSN:** 03126307

**Source type:** Newspapers

**Language of publication:** English

**Document type:** News

**ProQuest document ID:** 1080759102

**Document URL:**

[http://simsrad.net.ocs.mq.edu.au/login?url=https://search.proquest.com/docview/1](http://simsrad.net.ocs.mq.edu.au/login?url=https%3A//search.proquest.com/docview/1)

080759102?accountid=12219

**Copyright:** (Copyright (c) 2012 Fairfax Media Publications Pty Limited. [www.theage.com.au.](http://www.theage.com.au/) Not available for re-distribution.)

**Last updated:** 2017-11-19

**Database:** Australia &New Zealand Newsstream

# LINKS

Database copyright © 2020 ProQuest LLC. All rights reserved.

[Terms and Conditions](https://search.proquest.com/info/termsAndConditions) [Contact ProQuest](http://www.proquest.com/go/pqissupportcontact)
